# Attitudes towards priority setting in the Norwegian health care system: a general population survey

**DOI:** 10.1186/s12913-022-07806-9

**Published:** 2022-04-05

**Authors:** Carl Tollef Solberg, Eirik Joakim Tranvåg, Morten Magelssen

**Affiliations:** 1 Centre for Medical Ethics (CME), Institute of Health and Society, Faculty of Medicine, University of Oslo, Postbox 1130, Blindern 0318, Oslo, Norway; 2 Bergen Centre for Ethics and Priority Setting (BCEPS), Department of Global Public Health and Primary Care, Faculty of Medicine, University of Bergen, Bergen, Norway; 3 Centre for Cancer Biomarkers (CCBIO), Department of Global Public Health and Primary Care, Faculty of Medicine, University of Bergen, Bergen, Norway; 4 MF Norwegian School of Theology, Religion and Society, Oslo, Norway

**Keywords:** Attitude survey, Empirical ethics, General population, Legitimacy, Norway, Priority setting

## Abstract

**Background:**

In an ideal world, everyone would receive medical resources in accordance with their needs. In reality, resources are often scarce and have an alternative use. Thus, we are forced to prioritize. Although Norway is one of the leading countries in normative priority setting work, few descriptive studies have been conducted in the country. To increase legitimacy in priority setting, knowledge about laypeople’s attitudes is central. The aim of the study is therefore to assess the general population’s attitudes towards a broad spectrum of issues pertinent to priority setting in the Norwegian publicly financed health care system.

**Methods:**

We developed an electronic questionnaire that was distributed to a representative sample of 2 540 Norwegians regarding their attitudes towards priority setting in Norway. A total of 1 035 responded (response rate 40.7%). Data were analyzed with descriptive statistics and binary logistic regression.

**Results:**

A majority (73.0%) of respondents preferred increased funding of publicly financed health services at the expense of other sectors in society. Moreover, a larger share of the respondents suggested either increased taxes (37.0%) or drawing from the Government Pension Fund Global (31.0%) as sources of funding. However, the respondents were divided on whether it was acceptable to say “no” to new cancer drugs when the effect is low and the price is high: 38.6% somewhat or fully disagreed that this was acceptable, while 46.5% somewhat or fully agreed. Lastly, 84.0% of the respondents did not find it acceptable that the Norwegian municipalities have different standards for providing care services.

**Conclusion:**

Although the survey suggests support for priority setting among Norwegian laypeople, it has also revealed that a significant minority are reluctant to accept it.

**Supplementary Information:**

The online version contains supplementary material available at 10.1186/s12913-022-07806-9.

## Background

Health resources are limited, and no country can meet the total demand for beneficial health care for all its citizens. Moreover, in most countries, the healthcare *demand* is increasing more than a sustainable supply of *resources* permits [1], a phenomenon sometimes termed “the health gap”. There are at least three overall strategies for reducing the health gap. First, we can increase the total healthcare funding (i.e., *increase* resources). Second, we can reorganize the healthcare system to form a less expensive care level (i.e., *decrease* demand). Third, we can seek to prioritize scarce resources (i.e., to *seek a better balance* between resources and demand).

To set priorities involves “treat[ing] something as being more important than other things” [2]. Priority setting in health care can accordingly be defined as “the task of determining the priority to be assigned to a service, a service development or an individual patient at a given point in time” [3]. Setting priorities concerns therefore a rationing of available resources in order to achieve the most important things first. Sustainable priority setting requires legitimacy among the involved parties, and it is therefore relevant to assess the public’s opinion and attitudes on priority setting.

Thus, the aim of the study is to assess the general population’s attitudes towards a broad spectrum of issues pertinent to priority setting in the Norwegian publicly financed health care system. More specifically, this study surveys attitudes towards priority setting regarding (I) health financing, (II) financing for new and expensive cancer drugs, and (III) municipal services. Hypotheses were formulated in each of these three domains (Table 1).

**Table 1:** Six hypotheses about public attitudes to priority setting

Domain	Hypothesis #	Hypothesis
(I) Health financing	1	A majority thinks that public health funding should be increased
	2	Younger people more often than older people want to increase public health funding
(II) New cancer drugs	3	A majority accepts that new cancer drugs with poor cost-effectiveness and only moderate benefit are not used in the public health service
	4	When cancer drugs have at least a small possibility of curing, only a minority will accept that the drugs are not used in the public health service
(III) Municipal services	5	In a specific case, only a minority will accept that care is provided in a nursing home rather than the patient’s home in order to save money
	6	Only a minority will accept that service standards differ between municipalities

### The Norwegian health care system

The Norwegian health care system has a mixed financing model where all residents are covered for most medical and care services. It is mainly financed by taxes and supplemented by out-of-pocket payments. In 2019 the out-of-pocket share of total health expenditure was 13.9% [4]. Predominantly, two different sectors provide services: a *primary health care sector,* financed and organized by the 356 municipalities, and a *specialized health care sector*, financed and organized by regional health trusts and the Norwegian Ministry of Health and Care Services [5]. In such a public health insurance system, all Norwegian citizens can be considered legitimate stakeholders for priority setting.

In Norway, there is a well-established tradition for public discussions on priority setting and how best to conduct it. Since 1987, Norway has had five white papers [6–10] and one working group [11] on priority setting in the health care services. Norway employs three formal criteria to guide priority setting. First, according to *the health benefit criterion*, more priority is given to interventions with a higher expected benefit. Second, according to the *resource criterion*, more priority is given to interventions that require fewer resources. Third, according to the *severity criterion*, more priority is given to interventions that target more severe conditions. These three criteria must be considered together (rather than in isolation), and they apply on both a group and individual level [12]. What is more, these three criteria are enshrined in laws and regulations [13].

Throughout the Norwegian priority setting discourse, the issue of how to operationalize the severity criterion has been the crux. Internationally, there is a rich literature on how this might be accomplished [14]. Currently, the Norwegian severity criterion is operationalized as an *absolute shortfall* on a group level [15]. While the three criteria were mostly made for the specialized health care sector, a 2018 white paper [10] recommended that the same three criteria (with the added element of attention to ‘coping’) be also applied to the primary health care sector. This has resulted in yet another government white paper [16].

### The literature and gaps

There is a rich literature on stakeholders’ views regarding priority setting in health care. In Europe, several surveys have been conducted on how stakeholders view different criteria for priority setting [17–26]. Some use traditional questionnaires [17–20, 23, 26], while others apply discrete choice experiments (including vignettes) [21–24]. A handful of surveys have also explored the same question using qualitative methods [25, 28–30]. Furthermore, there exist surveys concerned with how physicians in Norway and other countries perceive priority setting [17, 19]. However, given Norway’s pioneering role in normative priority-setting work, relatively few descriptive studies have been conducted regarding Norwegian citizens’ attitudes towards priority setting in health care [31–33].

In 1998, a representative sample of 1 342 Norwegian laypeople was surveyed regarding their attitude towards central health political questions. This survey included a subgroup of questions regarding priority setting in health. The general response pattern showed skepticism towards healthcare rationing. This sample also showed that women were more reluctant towards health care rationing than men [31].

The largest study to date on attitudes to priority setting is a Norwegian Citizen Panel report from 2014. Important patterns presented in this report were as follows: that health is important to Norwegian citizens; that a majority of the respondents is willing to use a large share of public resources on health; that a non-trivial proportion of the respondents accepts that we cannot do all beneficial health interventions with the available resources; and that severe conditions should receive high priority beyond their cost-effectiveness [32]. Moreover, a recent study of the Norwegian media discourse on expensive cancer drugs is also worth mentioning. The pattern found in this media discourse is that cancer drugs are de facto expensive; have an indubitable efficacy; and that lifetime gained for a cancer patient is perceived as an absolute good [34].

Despite (apparent) political agreement within Norway about the overall approach, specific cases often lead to public controversy and significant attention [35]. Knowledge about citizens’ views and values in priority setting are crucial for both theoretical legitimacy and practical support for the current system. In order to be perceived as legitimate, actual priorities should not run counter to the population’s perception of what is just [30]. Specifically, we lack knowledge of how Norwegian laypeople perceive the different strategies for coping with the health gap and differences in treatment opportunities among different municipalities, as well as their attitudes towards the priority setting of expensive cancer treatment.

## Methods

### Questionnaire

A questionnaire was designed to test the six hypotheses (Table 1). In December 2019, the commercial firm Kantar distributed the electronic questionnaire via email to members of their nationally representative panel. Panel members were told that the questionnaire would assess attitudes towards ethical issues in healthcare. The questions on priority setting constituted one of four parts of the total questionnaire. Here, respondents were asked to take a stand on eight propositions. An English translation of the Norwegian questionnaire is available as an appendix in Additional file 1.

Question 1 (Q1) asked whether the funding of publicly financed health services should be reduced, kept the same as today, or increased somewhat or greatly. Q2 asked where any increase should come from. Q3–6 confronted respondents with propositions about the rationing of- and co-payment for expensive cancer drugs, with Q3 asking respondents to take a stand on the proposition, “It is acceptable that the state says “no” to new cancer drugs when the effect is small and the price is high”. Q4 stated that “It is acceptable that patients who can afford it can buy cancer drugs privately”, whereas Q5 said, “Cancer treatment should be prioritized higher than treatment of other diseases that are equally severe”.

Finally, Q6 involved a questionnaire experiment where respondents were randomized to receive one of three variations of a brief vignette. The vignette read, “Imagine that a new anti-cancer drug is launched. Treatment with the drug costs one million Norwegian Kroner (NOK) [approximately 100 000 Euros] per patient.” Group A received the following addition: “The average patient’s health benefit is two months prolonged survival”. Alternatively, Group B received, “The average patient’s health benefit is two months prolonged survival, but a minority, 5% of patients, become long term survivors.” Lastly, group C received the addition, “The average patient's health benefit is two months prolonged survival, but a minority, 15% of patients, become long term survivors”. All groups were then asked to take a stand on the same proposition (Q6): “It is acceptable that the state says “no” to pay for the new drug because the price is high compared to the effect.”

The final questions concerned priority setting in the primary health and care sector. Respondents were randomized to receive one of two cases and questions. The first randomized group received: “A patient with a severe neurological disease has become dependent on a ventilator (breathing machine). Without treatment, the patient dies. However, the treatment is very expensive for the municipality. The nursing staff is needed around the clock, and it costs approximately NOK 6 million annually. The treatment has so far been provided in the patient’s home, but the municipality now wants to move the treatment to the nursing home to save money.” Respondents were then asked to consider (Q7): “It is acceptable that the municipality only offers the treatment in the nursing home and not in the patient’s home, to save money. How much do you agree or disagree?”.

The second group received this case: “An elderly man with dementia (Alzheimer’s) lives in an apartment with his wife. He is ambulant but still dependent on supervision around the clock. He has help from home nursing care, but a lot nevertheless falls on his wife. An application is made for a permanent nursing home residence for the man, but the application is rejected. The justification is that it is safe to let the man stay at home as he does not live alone.” Respondents were then asked to consider (Q8): “It is acceptable that the municipality expects relatives to contribute to the man’s care.” Finally, all respondents were asked to consider (Q9): “It is acceptable that Norway’s municipalities have different standards for the provision of care and care services.” 

For Q3–9, respondents could select the options “Fully disagree”, “Somewhat disagree”, “Neither agree nor disagree”, “Somewhat agree”, “Fully agree”, and “Do not wish to state”.

### Population and statistical analyses

In all, 2 540 panel members were invited to respond to the questionnaire, out of which 1 076 responded. We received 1 035 complete responses (response rate 40.7%), which were weighted according to age, gender, and geographical region to achieve a closer match with national averages (See Table 1 in Online Appendix for the demographic characteristics of the participants). Analyses were performed on weighted data.

Statistical analyses were performed with IBM SPSS Statistics version 26. The results are presented with descriptive statistics and mean Likert scores on a five-point scale, with “fully disagree” (= 1) and “fully agree” (= 5) as scale anchors. For three key questions (Q1, Q3, Q4), binary logistic regression analyses were carried out to assess the influence of age, sex, education, and religious affiliation on attitudes. Here, age was dichotomized as below or above 45 years of age and education as college/university educated or not. The questionnaire was developed through discussions among the authors and reviewed by other colleagues. Lay-persons pilot tested the electronic version in two stages.

## Results

### Funding of the publicly financed health service

The majority of the respondents supported the view that the funding of publicly financed health services in Norway should be increased (Q1), with 39.0% preferring a somewhat increase and 33.9% a great increase. Only 2.9% preferred a reduction, and 21.2% preferred no change in healthcare funding. This confirmed hypothesis H1 (Table 1). Females and those less educated were more likely to prefer a great increase (Table 2); there were, however, no age differences, thus disconfirming H2. When asked where the increased funding should come from (Q2), 36.2% support an increase in taxes, while 30.1% want money to be spent from the Government Pension Fund Global (colloquially named “The Oil Fund”). Another 18.9% want money to be transferred from other sectors in society, while only 4.7% want to increase patient co-payments.

**Table 2:** Binary logistic regression analysis. Age, sex, education, and religion as independent variables

	**Q1 (Significantly increase public spending)**	**Q3 (Acceptability of rationing)**	**Q4 (Acceptability of co-payment)**
Age (> 45)	OR 0.80 (CI 0.61–1.07) NS	OR 0.77 (CI 0.58–1.01) NS	OR 0.66 (CI 0.50–0.87) p = 0.003
Sex (female)	OR 1.52 (CI 1.16–2.01) p = 0.003	OR 0.52 (CI 0.40–0.68) p < 0.001	OR 0.64 (CI 0.49–0.84) p = 0.001
Education (college/uni.)	OR 0.58 (CI 0.44–0.77) p < 0.001	OR 2.28 (CI 1.72–3.01) p < 0.001	OR 1.08 (CI 0.82–1.42) NS
Religious affiliation (Christianity)	OR 1.00 (CI 0.75–1.33) NS	OR 0.79 (CI 0.60–1.04) NS	OR 1.03 (CI 0.78–1.36) NS

OR = Odds ratio. CI = 95% confidence interval. NS = non-significant

### Financing for new and expensive cancer drugs

The respondents disagree about whether it is acceptable that Norwegian authorities decline to finance new cancer drugs with a high price and low effect (Q3, Table 3). While 47.3% either fully (18.8%) or somewhat agree (28.6%), a large proportion also fully (18.7%) or somewhat disagree (18.6%) that it is acceptable that the authorities say no to such drugs. Thus, although support for such rationing was substantial, it was held by a minority only – thus disconfirming H3. Males and the highly educated more often find it acceptable to say no (Table 2). A total of 57.9% of respondents fully or somewhat agree that it is acceptable that those who can afford it buy cancer treatment at private institutions (Q4). Here, young and male respondents more often find it acceptable. Few (17.5%) agree that cancer should receive a higher priority than other diseases (Q5), yet a third remain undecided on the issue.

**Table 3:** Respondents’ views on rationing, co-payment, and municipal services. N (%). N = 1035

	**Fully disagree**	**Somewhat disagree**	**Neither agree nor disagree**	**Somewhat agree**	**Fully agree**	**Unanswered**	**Mean Likert score**
Q3 (rationing cancer drugs)	193 (18.7)	193 (18.6)	131 (12.6)	296 (28.6)	194 (18.8)	28 (2.7)	3.10
Q4 (co-payment)	124 (11.9)	120 (11.6)	167 (16.1)	277 (26.7)	322 (31.1)	25 (2.5)	3.55
Q5 (prioritize cancer)	290 (28.1)	195 (18.8)	339 (32.8)	110 (10.7)	71 (6.9)	28 (2.8)	2.47
Q9 (municipal standards)	620 (59.9)	230 (22.3)	46 (4.5)	80 (7.8)	34 (3.3)	23 (2.3)	1.69

As expected, the proportion of respondents who think it is acceptable to say no to expensive cancer drugs decreases as the number of patients who can benefit increases (Q6, Table 4). The agreement that rationing would be acceptable decreased from two-thirds of respondents to slightly more than one-third when the prospect of long-term survival increased. H4 was thereby confirmed.

**Table 4:** Respondents’ views on the acceptability of rationing an expensive cancer drug with different prospects of survival (Q6). N (%)

	**Fully disagree**	**Somewhat disagree**	**Neither agree nor disagree**	**Somewhat agree**	**Fully agree**	**Unanswered**	**Mean Likert score**	**N**
2 months survival on average	28 (8.0)	27 (7.7)	45 (12.9)	126 (36.1)	109 (31.2)	14 (4.0)	3.78	349
+ 5% long-term survival	53 (15.1)	70 (19.9)	60 (17.0)	100 (28.4)	53 (15.1)	16 (4.6)	3.09	352
+ 15% long-term survival	71 (21.5)	78 (23.6)	49 (14.8)	90 (27.2)	30 (9.1)	13 (3.9)	2.78	331

### Municipal services

In the case vignette, 55.5% either fully or somewhat agree that it was acceptable for the municipality to save costs by only providing health care at a nursing home and not at home (Q7). Here, 27.5% disagree fully or somewhat. H5 was thus disconfirmed. Another rationing decision was less well-received: In Q8, only 20.8% consider it fully (3.0%) or somewhat (17.8%) acceptable that the municipality expects relatives to contribute to care; 70.7% disagree fully or somewhat.

When asked whether it is acceptable that Norwegian municipalities offer different standards for their health and care services (Q9), only a minority agree (Table 3). H6 was confirmed. Whether respondents had received one or the other preceding vignette (Q7 or Q8) did not influence attitudes (data not shown).

## Discussion

In this survey of a representative group of Norwegian citizens, we have found that a majority of the respondents support an increase in public health care funding. As for new cancer drugs, respondents are sensitive to the treatment’s health benefit: as a larger share of patients benefit from treatment, more respondents think that the authorities must provide the drug to all patients. A large majority of responders disapprove of Norway’s municipalities having different standards for the provision of care and care services.

### Funding of the publicly financed health service

That a majority of the respondents support increased funding of public health care services in Norway is a strong message to politicians and policymakers. Still, Norway’s healthcare spending is already among the highest globally, ranked as the third-highest spender by OECD in 2019, with a per-capita health care expenditure of $5 986 [2]. We also find it interesting that almost one-third of the responders prefer the increase in health spending financed from the Government Pension Fund Global. In contrast, a priority setting discourse usually takes for granted that health budgets are fixed. What is more, these results are in line with the 2014 survey by the Norwegian citizen panel, where respondents expressed a willingness to use a large share of public resources on health [32]. A willingness for increased public health care funding – relative to other public sectors – may suggest that many people conceive health as a *special* good [35, 36].

### Financing for new and expensive cancer drugs

Only a slight minority agree that it is acceptable that the authorities refuse to offer cancer drugs that provide low benefits at a high cost. Almost as many find it unacceptable. This pattern may illustrate the public controversy surrounding such reimbursement decisions. What is more, there is an apparent paradox in that only a small minority prefer increased co-payments to an increase in funding for publicly financed health services, while more than half of the respondents agree that patients should be allowed to buy cancer treatment from private providers. Such a response pattern may suggest that many people consider cancer exceptional as a category of disease or that the willingness to buy from private providers increases as people consider their conditions more *severe*. Alternatively, it may suggest that co-payments to increase funding are conceived differently from paying the entire cost of treatment to a private provider.

Interestingly, these response patterns are nuanced in our case vignette, where we find that the percentage of responders who find non-funding acceptable is sensitive to the drug’s benefit. As more patients are helped, fewer responders find a rejection acceptable. This pattern is in line with the intention of the Norwegian drug reimbursement system, where increased priority should be given to services that provide *more* health benefits [37]. Moreover, this response pattern is more nuanced than the aforementioned Norwegian media discourse attitudes concerning cancer and cancer drugs [34].

### Norway’s municipal services

A clear majority find inequalities in service standards between municipalities unacceptable. Such a response pattern leads to a dilemma because Norwegian municipalities are self-governing bodies with autonomy in decision-making and resource allocation. Even though patients are guaranteed a certain standard of services by law, municipalities have considerable freedom in how services are provided. Municipal autonomy is therefore in friction with the ideal of equal standards preferred by the respondents.

Arguably, this attitude also conflicts with how services are delivered in practice. The complexity of primary health care priority setting is detailed in an official Norwegian report published in 2018 [10], and further discussed in a Norwegian white paper from 2021 [16]. As mentioned, the three official Norwegian priority-setting criteria – health benefits, resources, and severity – were designed mostly in the context of the specialized health care system. However, there are many crucial asymmetries between Norwegian *specialist health care* and *primary health care*. Norwegian primary health care has broader goals; is more interwoven with wider society; has a weaker evidence base and longer timeframe; and is more concerned with care and prevention. Priority setting is a powerful tool to cope with unequal for health and care services standards. Accordingly, there seems to be at least an implicit will to prioritize in the Norwegian primary health care system.

### The value of legitimacy in priority setting

Performing surveys might contribute to legitimacy if findings are taken into account when priority setting decisions are made and the system is refined. Priority setting is concerned with authoritative choices in the ordering of goods. Legitimacy is concerned with people’s *actual* beliefs about something, with the *justification* of those beliefs, and a means to increase trust and compliance [38]. For priority setting in health, every citizen is a stakeholder. Eventually, we will all be in touch with our own countries’ health care system. The quality of the health care offered will, among other things, depend on the health budget and the priorities set within that budget.

### Relevance for priority setting

Our findings add relevant knowledge to the existing body of survey literature on priority setting preferences and may also strengthen legitimacy in priority setting decisions, as discussed above. In addition, they following may also be of more direct relevance. First, as health expenditures are projected to rise in most countries [2], the funding of- and priority setting in health care will be a key topic where our findings may be relevant. This may be particularly timely after the COVID-19 pandemic, where many health care systems have experienced a resource shortage. Second, how to finance expensive cancer drugs is a critical question heavily debated in the UK, US, and European Union. In Norway, the system for drug reimbursement (“New methods” system) has recently been evaluated, and our findings may inform the process. Third, our results may be relevant to municipalities and the primary health sector, as there is a lack of consistent priority setting practices compared to hospital settings and drug reimbursement processes.

### Strengths and limitations

Regarding the strengths, our study provides novel and valuable empirical knowledge regarding essential aspects of priority setting in the Norwegian public health care system. We have also been concerned with people’s attitudes toward changing the health budget, the value of consistency in the services offered, and people’s attitudes towards expensive cancer treatments. To our knowledge, the two latter topics have not been studied in the Norwegian context before.

However, our study is not without its limitations. The response rate of 40.7% is decent, yet a non-response bias cannot be excluded. Moreover, an attitude survey with fixed response alternatives will not provide insight into respondents’ reasoning processes or any further nuances in their views. For this, a qualitative approach would have been more appropriate.

## Conclusion

In conclusion, we have found a certain willingness to prioritize health care resources among Norwegian laypeople concerning expensive cancer treatment, certain municipality services, and health financing. This again suggests a willingness to conduct priority setting in a broad sense among the general population in Norway.

## Supplementary Information

**Additional file 1.** **Table 1. **Demographic characteristics of participants. *N*=1035

## Data Availability

The datasets generated and analyzed during the current study are not publicly available for reasons of practicality. However, the data are available upon reasonable request to the corresponding author.
