# Correction to: Attitudes towards priority setting in the Norwegian health care system: a general population survey

**DOI:** 10.1186/s12913-022-07994-4

**Published:** 2022-05-12

**Authors:** Carl Tollef Solberg, Eirik Joakim Tranvåg, Morten Magelssen

**Affiliations:** 1 Centre for Medical Ethics (CME), Institute of Health and Society, Faculty of Medicine, University of Oslo, Postbox 1130, Blindern, 0318 Oslo, Norway; 2 Bergen Centre for Ethics and Priority Setting (BCEPS), Department of Global Public Health and Primary Care, Faculty of Medicine, University of Bergen, Bergen, Norway; 3 Centre for Cancer Biomarkers (CCBIO), Department of Global Public Health and Primary Care, Faculty of Medicine, University of Bergen, Bergen, Norway; 4 MF Norwegian School of Theology, Religion and Society, Oslo, Norway

**Correction to: BMC Health Serv Res 22, 444 (2022)**

**https://doi.org/10.1186/s12913-022-07806-9**

Following publication of the original article [1], an error was identified in the Reference list. Due to a typesetting mistake, reference 16 was retained with invalid information, instead of removing it.

The original article [1] has been corrected: the original reference 16 has been removed from the Reference list and the subsequent references and their citations have been renumbered. The publisher apologises to the authors and readers for the inconvenience caused by this error.
