# Supplementary material for: Attitudes towards priority setting in the norwegian health care system: a general population survey
Source: BMC Health Serv Res. 2022 Apr 5;22:444. doi: 10.1186/s12913-022-07806-9 (PMC8980508; doi:10.1186/s12913-022-07806-9)
Supplement: Supplementary file 1 — Additional file 1. Table 1. Demographic characteristics of participants. N=1035 [file 12913_2022_7806_MOESM1_ESM.docx]

**Online appendix**

**Table 1.** Demographic characteristics of participants. N=1035

|  | Unweighted (N (%)) | Weighted (N (%)) | National averages (%; from SSB.no) |
| --- | --- | --- | --- |
| Age  Mean  Under 30  30-44  45-59  60+ | 53.5 years  125 (12.1)  188 (18.2)  291 (28.1)  431 (41.6) | 47.8 years  210 (20.3)  266 (25.7)  266 (25.7)  293 (28.3) | 19.4  25.4  25.4  29.8 |
| Female | 534 (51.6) | 514 (49.6) | 49.8 |
| Highest completed education  Primary school  Upper secondary school  Vocational school  College/university <5 years  College/university ≥5 years | 48 (4.6)  269 (26)  95 (9.2)  369 (35.7)  254 (24.5) | 39 (3.8)  296 (28.6)  84 (8.1)  361 (34.9)  255 (24.7) |  |
| Worldview  Christian  Muslim  Other religion  Non-religious  Unanswered | 533 (51.5)  6 (0.6)  13 (1.3)  420 (40.6)  63 (6) | 485 (46.8)  9 (0.9)  16 (1.5)  462 (44.7)  63 (6.1) |  |

***English translation of the Norwegian questionnaire**

*

What is your view on the following: Funding of publicly financed health services should (…)?

(Be reduced – Be kept same as today – Be increased somewhat – Be increased greatly – Do not wish to state)

If funding for the publicly financed health services were to be increased, where should the money come from?

(From increased tax income – From other societal sectors – From increased patient co-payment – From the Norwegian Oil Fund [colloquial name for the Norwegian Government Pension Fund] – Funding should not increase – Do not want to state)

Some new cancer drugs are not introduced into the publicly financed health services because the average effect is considered not good enough and the price is too high. This opens up a private market where those who can afford it can buy the drugs themselves. How much do you agree or disagree with the following statements?

It is acceptable that the state says “no” to new cancer drugs when the effect is small and the price is high.

(Fully disagree – Somewhat disagree – Neither agree nor disagree – Somewhat agree – Fully agree – Do not wish to state)

It is acceptable that patients who can afford it can buy cancer drugs privately

(Fully disagree – Somewhat disagree – Neither agree nor disagree – Somewhat agree – Fully agree – Do not wish to state)

Cancer treatment should be prioritized higher than treatment of other diseases that are equally severe.

(Fully disagree – Somewhat disagree – Neither agree nor disagree – Somewhat agree – Fully agree – Do not wish to state)

Imagine that a new anti-cancer drug is launched. Treatment with the drug costs one million Norwegian Kroner (NOK) [approximately 100 000 Euros] per patient.

*Randomized to receive one of three additions:*

A) The average patient's health benefit is two months prolonged survival

B) The average patient's health benefit is two months longer survival, but a minority, 5% of patients, become long term survivors.

C) The average patient's health benefit is two months longer survival, but a minority, 15% of patients, become long term survivors.

It is acceptable that the state says "no" to pay for the new drug because the price is high compared to the effect. How much do you agree or disagree with this?

(Fully disagree – Somewhat disagree – Neither agree nor disagree – Somewhat agree – Fully agree – Do not wish to state)

*Randomized to receive one or two cases/questions:*

1) A patient with a severe neurological disease has become dependent on a ventilator (breathing machine). Without treatment, the patient dies. However, the treatment is very expensive for the municipality. The nursing staff is needed around the clock, and it costs approx. NOK 6 million annually. The treatment has so far been provided in the patient’s home, but the municipality now wants to move the treatment to the nursing home to save money. It is acceptable that the municipality only offers the treatment in the nursing home and not in the patient's home to save money. How much do you agree or disagree?

(Fully disagree – Somewhat disagree – Neither agree nor disagree – Somewhat agree – Fully agree – Do not wish to state)

2) An elderly man with dementia (Alzheimer's) lives in an apartment with his wife. He is ambulant but still dependent on supervision around the clock. He has help from home nursing care, but a lot nevertheless falls on his wife. An application is made for a permanent nursing home residence for the man, but the application is rejected. The justification that it is safe to let the man stay at home as long as he does not live alone. It is acceptable that the municipality expects relatives to contribute to the man’s care’. How much do you agree or disagree?

(Fully disagree – Somewhat disagree – Neither agree nor disagree – Somewhat agree – Fully agree – Do not wish to state)

It is acceptable that Norway's municipalities have different standards for the provision of care and care services. How much do you agree or disagree?

(Fully disagree – Somewhat disagree – Neither agree nor disagree – Somewhat agree – Fully agree – Do not wish to state)
